# Detection of spring viraemia of carp virus in imported amphibians reveals an unanticipated foreign animal disease threat

**DOI:** 10.1038/emi.2016.94

**Published:** 2016-09-07

**Authors:** Hon S Ip, Jeffrey M Lorch, David S Blehert

**Affiliations:** 1 US Geological Survey – National Wildlife Health Center, Madison, WI 53711, USA

**Keywords:** host range, pathogen introduction, wildlife trade

## Abstract

Global translocation of plants and animals is a well-recognized mechanism for introduction of pathogens into new regions. To mitigate this risk, various tools such as preshipment health certificates, quarantines, screening for specific disease agents and outright bans have been implemented. However, such measures only target known infectious agents and their hosts and may fail to prevent translocation of even well-recognized pathogens if they are carried by novel host species. In a recent example, we screened an imported shipment of Chinese firebelly newts (*Cynops orientalis*) for *Batrachochytrium salamandrivorans*, an emergent fungal pathogen of salamanders. All animals tested negative for the fungus. However, a virus was cultured from internal organs from 7 of the 11 individual dead salamanders and from two pools of tissues from four additional dead animals. Sequencing of a portion of the glycoprotein gene from all viral isolates indicated 100% identity and that they were most closely related to spring viraemia of carp virus (SVCV). Subsequently, SVCV-specific PCR testing indicated the presence of virus in internal organs from each of the four animals previously pooled, and whole-genome sequencing of one of the viral isolates confirmed genomic arrangement characteristic of SVCV. SVCV is a rhabdovirus pathogen of cyprinid fish that is listed as notifiable to the Office International des Epizooties. This discovery reveals a novel route for potential spillover of this economically important pathogen as rhabdovirus has not previously been documented in amphibians.

## Introduction

Emerging infectious diseases (EIDs) of wildlife represent a major threat to global biodiversity.^1^ Although mechanisms behind EIDs are multifactorial, many large-scale wildlife population declines attributed to disease are the result of anthropogenic translocation of infectious agents.^2^ Such introductions have led to precipitous declines of North American bat populations from white-nose syndrome,^3^ endangerment of wild mammalian carnivore species by canine distemper,^4^ and local and global extinctions of amphibians owing to chytridiomycosis.^5^ As a more recent example, introduction of *Batrachochytrium salamandrivorans* (*Bsal*) to Europe is causing catastrophic declines of native salamander species.^6^ This emergent fungal pathogen is believed to have reached Europe through international trade in wild Asian salamanders,^6^ underscoring another instance where movement of animals has facilitated translocation of associated pathogens.

Recently published assessments of the global risk that *Bsal* poses to amphibians indicates that introduction of this pathogen to North America, home to the world's greatest diversity of salamander species, could have catastrophic impacts on these wild populations.^7, 8^ To further evaluate the risk for introduction of *Bsal* through legally imported amphibians, we screened salamanders imported from China into the United States for the fungus. The animals did not harbor *Bsal* but rather tested positive for spring viraemia of carp virus (SVCV), a pathogen not previously known to infect amphibians. Inadvertent introduction of SVCV, the cause of a reportable foreign animal disease in the United States, would present an economic threat to the commercial cyprinid aquaculture industry. Overall, this example highlights the challenges of reducing risks for translocation of pathogens when knowledge of host–pathogen relationships is incomplete.

## Materials and Methods

### VIRUS ISOLATION AND CHARACTERIZATION

Eleven Chinese firebelly newt (*Cynops orientalis*) carcasses that were part of a larger shipment of live newts imported to the United States from Hong Kong, China in August 2015 were subjected to virus culture to screen for ranavirus, a major pathogen of amphibia.^9^ Pools of sterilely collected organs from dead salamanders, including liver, kidney and spleen, were homogenized in Dulbecco's minimal essential medium in a 1:10 (weight to volume) ratio and inoculated into either epithelioma papulosum cyprini (EPC^10^) or zebrafish (ZF4^11^) epithelial cell monolayers. A different set of dissection instruments was used for each animal. Cultures were incubated at 10 °C–16 °C and examined for cytopathic effects (CPE) daily for 7–10 days. Cultures that did not exhibit CPE were blind passed at least once before being classified as negative. Cultures that exhibited CPE were frozen at −80 °C and thawed three times, and cellular debris was removed by centrifugation at 1000 *g* for 30 min at 4 °C. For electron microscopic analysis, aliquots of the supernatant were re-centrifuged at 100 000 *g* for 10 min in a Beckman Airfuge (lndianapolis, CA, USA). The pelleted viral particles were negatively stained with 2% phosphotungstic acid and examined in a Hitachi H-7600 transmission electron microscope (Schaumnberg, lL, USA).

For reverse transcription-PCR (RT-PCR) analysis, aliquots of the initial low-speed supernatant were extracted using the following kits according to the manufacturer's instructions: Qiagen DNeasy Blood and Tissue Kit (Valencia, CA, USA) (for DNA) or Ambion MagMAX Viral RNA Isolation Kit (Waltham, MA, USA) (for RNA). Primer SVCV F1 (5′-TCT TGG AGC CAA ATA GCT CAR RTC-3′) and SVCV R2 (5′-AGA TGG TAT GGA CCC CAA TAC ATH ACN CAY-3′) were used in a 25-μL one-step RT-PCR reaction based on Stone *et al.*^12^ using Qiagen One-Step reagents with 5 μL of RNA according to the manufacturer's instructions. Cycle conditions were as follows: 50 °C for 20 min and 95 °C for 10 min; 45 cycles of 95 °C for 30 s, 55 °C for 30 s, and 72 °C for 1 min. Reaction products (5 μL) were examined using a 1% agarose gel, samples with the expected amplicon size (714 bp) were sequenced with the PCR primers and the resulting sequence was characterized by BLAST analysis. A real-time RT-PCR (rRT-PCR) assay targeting the G gene was used to determine individual infection status of animals whose tissues had originally been pooled for virus isolation.^13^

### WHOLE-GENOME SEQUENCING AND PHYLOGENETIC ANALYSIS OF SVCV

Total RNA was prepared from cell cultures that were positive for SVCV using the Ambion MagMAX Viral RNA Isolation Kit according to the manufacturer's instructions. Samples were sequenced at the National Veterinary Services Laboratories (Ames, IA, USA) on an Ion Torrent PGM instrument (Life Technologies, Carlsbad, CA, USA) using Ion Total RNA-Seq (v2) chemistries as per the manufacturer's instructions. Sequencing reads were assembled against reference sequences (GenBank KJ513477 and DQ491000) selected from results of BLAST analysis of a 2500-nt fragment generated by Sanger sequence analysis^14^ of isolate salamander/USA/202238/2015 (hereafter 202238). The assembled sequence has been deposited in GenBank (accession KU230365).

Representative nucleotide sequences from SVCV were aligned using MUSCLE^15^ and the best-fit model of nucleotide substitution was determined using MEGA 7.0.^16^ Median joining network analysis was performed on the aligned sequences using Population Analysis with Reticulate Trees (PopART, http://popart.otago.ac.nz) with epsilon=0. The network was recalculated 1000 times. Where not specified, default parameters were used. The aligned sequences were also analyzed using maximum likelihood by RAxML-HPC2 8.2.4 with 1000 bootstraps and GTRCAT model used during bootstrapping.^17^ Sequences were additionally compared by Bayesian analysis using MrBayes 3.1.2^18^ employing an HKY model with gamma distribution and invariant sites. The analysis was performed for 1 000 000 generations (with 25% burn-in) and sampled every 10 000 generations. Where not specified, default parameters were used.

### HOST GENOTYPING

Recent re-evaluation of *Cynops orientalis* has resulted in the discovery of cryptic species.^19, 20^ To confirm the species of salamander that were part of the imported shipment, DNA was extracted from pooled organs (muscle tissue from tail, heart, tongue and stomach) of a subset of animals (*n*=4) using the Gentra Puregene Tissue Kit (Qiagen) according to the manufacturer's instructions. A portion of the mitochondrial NADH dehydrogenase subunit two gene (and adjacent tRNAs) was amplified with primers 3787F: 5′-TCG TGC ACC CAC TAC ACT AC-3′ and 5081R: 5′-GTC GTA GGG TCA AAG CCT GC-3′ (primer 3787F was modified slightly from Wu *et al.*^21^ to better match existing sequence data for *C. orientalis*). PCR amplification was conducted in 50-μL reaction volumes using GoTaq Flexi DNA polymerase (Promega, Madison, WI, USA) and 1 μL of DNA template that had been diluted 100-fold. Cycling conditions were as follow: 94 °C for 2 min; 35 cycles of 94 °C for 30 s, 52 °C for 45 s, and 72 °C for 90 s; and a final extension for 5 min at 72 °C. Amplicons were sequenced using the amplification primers and internal sequencing primer 4416F: 5′-ATA GCA TAC TCA TCC ATT GCA CA-3′.^21^ Chromatograms of the resulting sequences were viewed and edited (as necessary) in Lasergene 5.0 (DNASTAR, Madison, WI, USA). Final sequences were aligned with existing DNA sequences in GenBank, primarily following taxon sampling by Wu *et al.*^19^ The alignment was conducted using MAFFT^22^ through the online program GUIDANCE^23, 24^ with the following settings: 100 bootstrap repeats, 1000 maximum iterations, and ‘globalpair' pairwise alignment method. Phylogenetic analyses using maximum likelihood and Bayesian methods were conducted on the final alignment with the programs RAxML-HPC2 version 8.2.4^17^ and MrBayes version 3.2.6,^18^ respectively, using the CIPRES Science Gateway.^25^ For both analyses, the general time reversible model with gamma distribution was used. For maximum likelihood analysis, the number of bootstrap iterations was set at 1000; all other RAxML parameters were left as default. For the Bayesian analysis, the number of generations was increased to 5 000 000 and remaining settings were default.

## Results

### VIRUS ISOLATION

**Figure 1:**
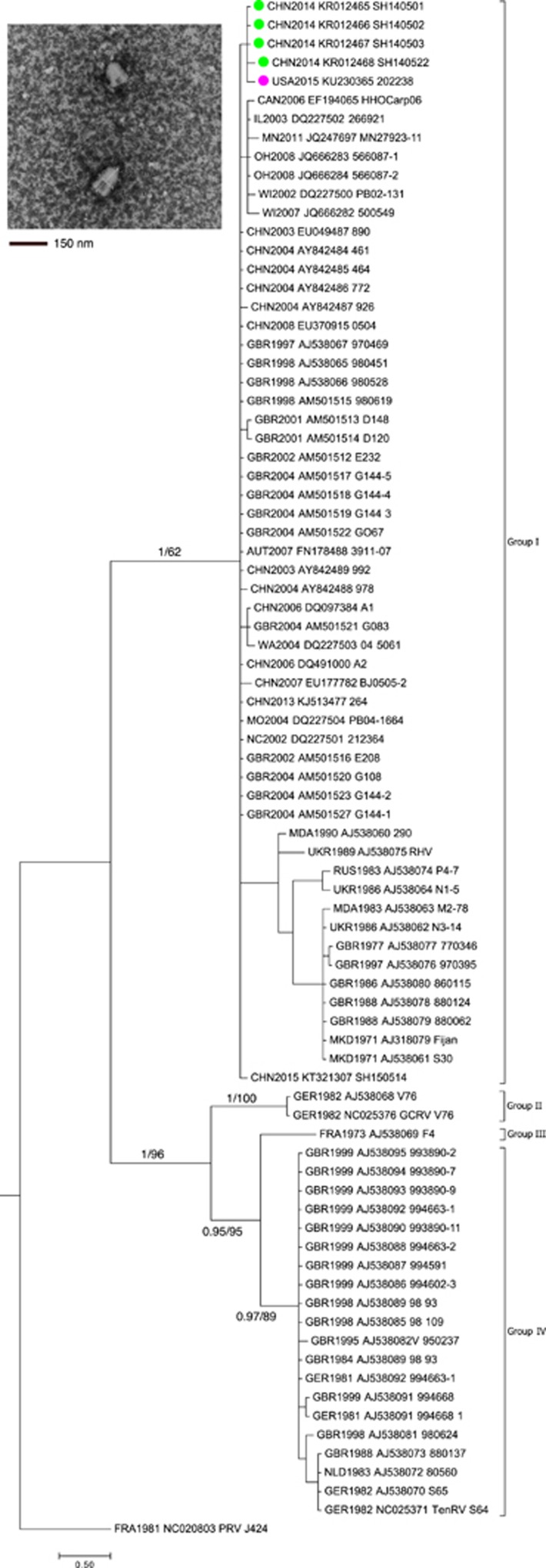
Phylogenetic tree from Bayesian and maximum likelihood analysis of nucleotide sequences of glycoprotein (G) gene from representative genogroups of spring viraemia of carp virus (SVCV). The tree is rooted on the distantly related perch rhabdovirus. Strains are designated by the first three letters corresponding to the ISO 3166-1 country code or the state abbreviation if the strain was isolated in the United States, the year, the GenBank accession number and the strain name. The salamander isolate of SVCV is labeled with a pink circle while the most closely related Chinese isolates of SVCV are labeled with green circles. Brackets enclose strains classified into genogroups according to Stone *et al.*^10^ Support at nodes are shown when statistically significant (Bayesian posterior probabilities/maximum likelihood bootstrap values). Negatively stained electron micrograph of the salamander SVCV. Scale bar: 150 nm.

CPE indicative of viral replication were observed in cell culture from nine samples representing tissues from all 11 animals; two of the samples each consisted of pooled organs from 2 separate salamanders while the remaining seven samples were from individual animals (Table 1). Ranavirus was not identified. However, a bullet-shaped virus characteristic of rhabdovirus (Figure 1) was observed by transmission electron microscopic analysis of a representative isolate (202238). Based on the observed morphology, PCR testing was conducted on this isolate using a rhabdovirus-specific assay targeting the polymerase gene.^26^ An amplicon of the expected size (260 nt) was generated, and sequence analysis showed closest similarity with the genus *Sprivivirus* in the family *Rhabdoviridae* (99% identity with SVCV A1, GenBank DQ097384). All four animals previously pooled for virus isolation purposes were individually positive using a SVCV-specific rRT-PCR test (Table 1).^13^

### CHARACTERIZATION OF THE SALAMANDER ISOLATES

**Figure 2:**
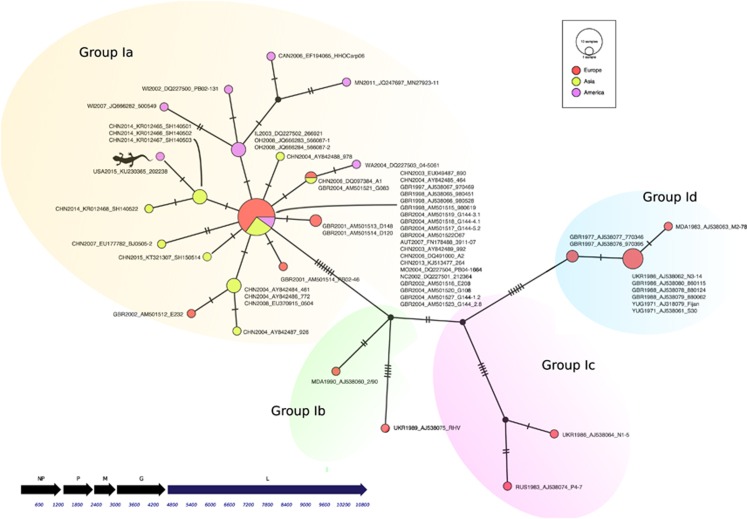
Genomic organization of salamander spring viraemia of carp virus (SVCV) and median joining phylogenetic network analysis of genogroup I viruses. The median joining network was constructed from the glycoprotein (G) gene. Each unique sequence is represented by a circle, and the size of each circle is proportional to the frequency of the sequence in the data set. Continent of origin for each isolate is coded as a pie chart with European isolates in red, Asian isolates in green and North American isolates in purple. Hatch marks represent genetic distance. Ovals are drawn around isolates that represent genogroups Ia, Ib, Ic and Id. Strains are designated with the first three letters corresponding to the ISO 3166-1 country code or the two-letter state abbreviation if the strain was isolated in the United States, the year, the GenBank accession number and the strain name. The salamander SVCV isolate is also labeled with a silhouette of a salamander. Schematic diagram of the genomic organization of salamander SVCV. Predicted coding regions of the nucleoprotein (NP), phosphoprotein (P), matrix (M), glycoprotein (G) and large polymerase (L) genes are represented.

To further characterize the nine viral isolates, a 714-nt fragment of the viral glycoprotein (G) gene was amplified by PCR.^12^ The amplified G gene fragments exhibited 100% identity to one another over the region sequenced. One viral isolate (202238) was also chosen for whole-genome sequencing. A near complete contig of 10 991 nt that encoded five genes (nucleoprotein, N; phosphoprotein, P; matrix, M; glycoprotein, G; and large polymerase, L) in an order that is characteristic of the genus *Sprivivirus* was obtained following assembly of the genome (GenBank accession KU230365, Figure 2). There was no nonvirion gene at the G–L junction as would be characteristic of vesiculoviruses and perhabdoviruses. The conserved transcriptional start signal of 3′-UUGUC-5′ and termination/polyadenylation signal of 3′-AUA CUU UUU UU-5′ were both present in all five genes, and their presence is characteristic (although not exclusively) of *Sprivivirus*.^27^ Overall, the salamander virus was assigned to SVCV in accordance with the International Committee on Taxonomy of Viruses (ICTV).^27^

Bayesian and maximum likelihood phylogenetic analyses of the G gene fragments indicated that the salamander isolates clustered most closely with SVCV genogroup I (Figure 1). Median-joining network analysis further classified the virus to genogroup Ia (Figure 2). The salamander SVCV was distinct from previous isolates of SVCV from North America but was closely related to recent isolates from China. Although relatively few whole-genome sequences for SVCV are available for comparison, the salamander SVCV protein coding regions (N, P, M, G and L) were all closely related (98.2%–99.8% identical) at the nucleotide and amino-acid levels to corresponding genes in SVCV strain SH140501 (Table 2), which was isolated from a goldfish in Shanghai sampled in April 2014.^28^ This finding further supported an Asian origin for the salamander SVCV isolates.

### PHYLOGENETIC ANALYSIS OF HOST SPECIES

**Figure 3:**
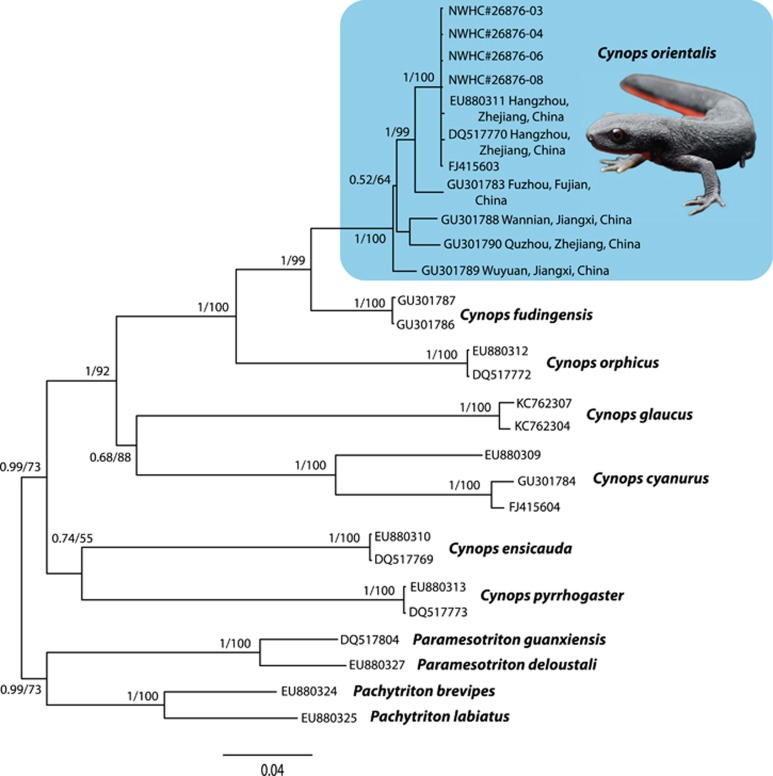
Phylogenetic tree from Bayesian analysis of nucleotide sequences from *Cynops* mitochondrial NADH dehydrogenase subunit two genes (and adjacent tRNAs) using the sampling scheme of Wu *et al.*^19^ The same tree topology was obtained by maximum likelihood analysis. Salamanders examined in this study (denoted as samples with the prefix NWHC#) resided within the *C. orientalis* clade. When known, locality information for *C. orientalis* is provided. The tree is rooted by salamanders of the genera *Pachytriton* and *Paramesotriton*. Support at nodes represent posterior probabilities (Bayesian)/bootstrap values (maximum likelihood).

To confirm the species identity of the salamanders infected with SVCV, a portion of the host mitochondrial NADH dehydrogenase subunit two gene (and adjacent tRNAs) was sequenced from a subset of four animals. The portion of DNA analyzed was 100% identical between the four animals examined. A representative sequence was deposited in GenBank (NWHC#26876-003; GenBank accession number KU647189). Phylogenetic analyses resulted in trees with the same topology and were similar to those of Wu *et al.*^19^ The salamanders that were positive for SVCV were part of a well-supported clade (Bayesian analysis: 100% posterior probability, maximum likelihood analysis: 100 bootstrap support; Figure 3) representative of *C. orientalis*. Specifically, imported salamanders grouped (Bayesian analysis: 100% posterior probability, maximum likelihood analysis: 100 bootstrap support) with *C. orientalis* that originated from the vicinity of Hangzhou, Zhejiang province, China, suggesting that the infected salamanders may have been collected from wild populations in that area.^19^

## Discussion

SVCV is a finfish rhabdovirus of major economic importance that is reportable to the Office International des Epizooties.^10^ The virus can be particularly devastating for the carp aquaculture industry and has also been reported to infect other commercially important species, including sheatfish (*Silurus glanis*), and possibly rainbow trout (*Oncorhynchus mykiss*) and tilapia (*Sarotherodon niloticus*).^10^ Based partly upon serological characterization, SVCV was formerly classified in the genus *Vesiculovirus*, family *Rhabdoviridae*, order *Mononegavirales* along with pike fry rhabdovirus (PFRV) and vesicular stomatitis virus, the type species of the genus. In 2012, most strains of PFRV and SVCV along with tench rhabdovirus (TenRV) and grass carp rhabdovirus (GrCRV) were proposed to be moved into a new genus, *Sprivivirus*, based on characterization of partial regions of individual gene sequences;^27^ this proposal was accepted in the current edition of the ICTV virus taxonomy.^29^

We determined that the salamander isolates represented SVCV based upon sequence analysis of the G gene. Although this classification scheme is still in use, additional finer-scale differentiation of SVCV genogroups has been proposed by Miller *et al.*^30^ and Xiao *et al.*^31^ based on the P and M genes, respectively. Our analyses focused on the G gene as this region is the most variable in the genome, it is the most consistently available sequence from isolates of SVCV, and its use conforms with current taxonomic criteria of the ICTV.^27^ Genomic organization and sequence analysis of the other genes of the salamander isolates also supported this taxonomic placement.

SVCV is additionally divided into four genogroups based on the sequence of a 550*-*nt region of the G gene. Genogroup I contains classic SVCV isolates; genogroup II contains isolates of GrCRV; and genogroups III and IV contain PFRV and TenRV, respectively. Genogroup I is further divided into genogroups Ia, Ib, Ic and Id.^12^ Genogroup Ia contains SVCV isolates from Asia and strains introduced into Europe and North America; genogroup Ib contains isolates of SVCV from eastern Europe; genogroup Ic contains isolates of SVCV from Russia and additional isolates from eastern Europe; and genogroup Id contains isolates of SVCV from western Europe. The salamander SVCV isolates are members of genotype Ia according to Bayesian and maximum likelihood (Figure 1) and network (Figure 2) phylogenetic analyses of the G gene. Moreover, the salamander SVCV is more closely related to recently characterized SVCV strains from Chinese cyprinids than to SVCV isolates previously identified in North America.

SVCV has been detected in Europe, Southeast Asia and the Middle East.^12^ It was first detected in the United States in 2002, in both farmed and wild fish populations experiencing mortality events.^32, 33^ Despite screening for SVCV and regulating live fish imported into the United States since 2006, new detections continue to be reported. To date, SVCV has been detected nine times in the United States and Canada,^34^ and epidemiological investigation and genetic analysis indicate that there may have been at least three independent introductions of SVCV into North America from as yet unidentified sources.^30^ Although the isolates of SVCV from salamanders described herein are distinct from viruses detected previously in North America, and their infectivity in fish has not yet been determined, this discovery suggests the possibility that introductions of SVCV could be due to movement of uncharacterized host species, such as amphibians.

Phylogenetic analyses confirmed the identity of imported salamanders infected with SVCV as *C. orientalis* and indicated that the animals likely originated from the vicinity of Hangzhou, Zhejiang province, China^19^ (Figure 3). Phylogenetic relatedness between the salamander SVCV and goldfish SVCV strain SH140501 isolated from Shanghai additionally supports the likelihood that the imported salamanders originated from this region of eastern China as Shanghai is 165 km from Hangzhou.^28^ We do not, however, know whether the salamanders harbored SVCV at the time of capture or were exposed to the virus during holding and transport. Moreover, it is unclear at this time, if the salamanders were infected by ingestion of or through contact with (perhaps at sites of dermal abrasions) contaminated materials. Nonetheless, these findings demonstrate the potential for translocated amphibians to serve as a source for the introduction of SVCV. Specifically, hundreds of thousands of *C. orientalis* are collected each year in China, and many of these animals enter the United States pet trade, creating the possibility that some individuals could escape or be released into the wild.^35^ Such releases, even in the absence of exotic salamander species becoming established, could represent a possible mechanism by which SVCV or other pathogens are spread. Furthermore, identification of *C. orientalis* as a host for SVCV raises the question of whether additional amphibian species imported into the United States could cryptically harbor pathogens of economic importance.

The susceptibility of amphibians worldwide to SVCV is not known but of particular concern is whether SVCV could act as a potential pathogen in already imperiled amphibian populations. Although the dead salamanders we examined were in good body condition, freezing and thawing of carcasses confounded attempts to observe lesions during histopathological examination. Thus we could not ascertain whether SVCV contributed to mortality or occurred without causing clinical signs or lesions. Indeed, non-lethal or asymptomatic SVCV infections have been documented in some wild and captive fish populations.^14, 36^ If, however, *C. orientalis* co-evolved with SVCV and can be infected without apparent detriment, this detection may further implicate Southeast Asia as the region from which this SVCV originated. Under such a scenario, salamanders from other parts of the world could represent potentially naive hosts subject to developing more severe disease upon exposure to this virus.

Preventing translocation of infectious pathogens through anthropogenic activities, including the global pet trade, has the potential to reduce the frequency and magnitude of EIDs that can affect human health, food security and global biodiversity. However, the scale of legal trade in wildlife species alone was valued at $323 billion USD in 2009,^37^ and mitigation of unintended global translocations of exotic pathogens will require a multifaceted approach. An example of one strategy is the recently imposed ban on importation and interstate transport within the United States of salamander species that may harbor the fungal pathogen *Bsal* (50 CFR Part 16). Recognition of the serious threat presented by *Bsal* and actions taken to prevent this invasive pathogen from reaching North America represent a substantial advance in proactive response to EIDs. Although the 11 newt carcasses and 75 skin swabs collected from live newts in the same shipment were negative for *Bsal*,^38^ discovery of SVCV, a major finfish pathogen in an imported amphibian species, provides further support for the importation ban. However, such restriction can be controversial, having economic, political, conservation and cultural impacts. Furthermore, as demonstrated by this study in which viable SVCV was identified in a novel order of vertebrate hosts, banning translocation of host species known to harbor a certain pathogen does not guarantee that a different host will not harbor the same pathogen. Similarly, bans targeting pathogens of concern may fail to prevent the movement of cryptic infectious agents that are currently unrecognized because they do not cause disease in hosts within their native ranges. In such cases, agent-agnostic diagnostic tools, such as virus isolation and Next-Generation Sequencing, may be necessary to provide comprehensive pathogen surveillance. Coordinated surveillance systems together with increased understanding of potentially novel pathogens offer the ability to augment importation restrictions by facilitating early detection and eradication of pathogens should they be introduced. Overall, restrictions on movement of some host species, active surveillance, quarantine practices, screening for known pathogens, basic research aimed at identifying possible pathogens before they emerge and educational campaigns emphasizing potential risks associated with release of invasive species can together underpin an international strategy to reduce risks of pathogen introduction.

## Figures and Tables

**Table 1:** Summary of spring viraemia of carp virus (SVCV) testing status for tissues from individual Chinese firebelly newts

**Animal ID**	**SVCV isolate**	**rRT-PCR**
26876-001*	Yes	Not applicable
26876-002	Yes	Not applicable
26876-003	Yes; pooled with 26876-004	Positive
26876-004	Yes; pooled with 26876-003	Positive
26876-005	Yes; pooled with 26876-006	Positive
26876-006	Yes; pooled with 26876-005	Positive
26876-007	Yes	Not applicable
26876-008	Yes	Not applicable
26876-009	Yes	Not applicable
26876-010	Yes	Not applicable
26876-011	Yes	Not applicable

Abbreviations: Real-time reverse transcription-PCR, rRT-PCR; spring viraemia of carp virus, SVCV. Individual animals are specified by their identity number (Animal ID); SVCV confirmation of isolate (SVCV Isolate: Yes or No); and SVCV rRT-PCR test status of tissues from animals that were previously pooled for virus isolation (Positive or Not applicable). Viral isolates were each identified by PCR testing and sequencing of a portion of the G-gene. An asterisk indicates the animal from which the entire genome of SVCV isolate 202238 was sequenced.

**Table 2:** The relatedness of the salamander spring viraemia of carp virus (SVCV) compared with Chinese SVCV strain SH140501 isolated from a goldfish in Shanghai in 2014

**Gene**	**% Nucleotide identity**	**% Amino-acid identity**
NP	98.9	99.3
P	98.8	98.4
M	98.5	98.7
G	98.2	98.4
L	99.0	99.8

Abbreviations: Glycoprotein, G; large polymerase, L; matrix, M; nucleoprotein, NP; phosphoprotein, P. Percentage of identity of the two strains at the nucleotide and amino-acid levels are presented.
